# External validation of the European association of urology biochemical recurrence risk groups to predict mortality after radical prostatectomy or radiation therapy in a North American cohort

**DOI:** 10.1002/bco2.70241

**Published:** 2026-06-29

**Authors:** Carlo Silvani, Alfonso Santangelo, Alex Stephens, Jack Considine, Shane Tinsley, Bassel Salka, Akshay Sood, Sebastiano Nazzani, Alberto Briganti, Andrea Salonia, Francesco Montorsi, Nicola Nicolai, Emanuele Montanari, Craig Rogers, Firas Abdollah

**Affiliations:** ^1^ VUI Center for Outcomes Research, Analysis and Evaluation Henry Ford Health System Detroit Michigan USA; ^2^ Urology Unit Fondazione IRCCS Istituto Nazionale dei Tumori Milan Italy; ^3^ Department of Clinical Sciences and Community Health University of Milan Milan Italy; ^4^ Division of Oncology, Unit of Urology IRCCS Ospedale San Raffaele, Vita‐Salute San Raffaele University Milan Italy; ^5^ Public Health Sciences Henry Ford Health System Detroit Michigan USA; ^6^ Department of Urology, The James Cancer Hospital and Solove Research Institute The Ohio State University Wexner Medical Center Columbus Ohio USA; ^7^ Department of Urology Fondazione IRCCS Ca' Granda, Ospedale Maggiore Policlinico Milan Italy

**Keywords:** neoplasm recurrence, local, prostatectomy, prostatic neoplasms, radiotherapy, risk assessment

## Abstract

**Objectives:**

This study aimed to externally validate the European Association of Urology (EAU) biochemical recurrence (BCR) risk stratification in a North American population after radical prostatectomy (RP) and radiation therapy (RT), where validation remains lacking despite prior European and Asian validation.

**Materials and methods:**

We identified all patients with BCR after RP or RT between 1995 and 2023 from a North American institutional database and classified them by EAU criteria. Primary outcome was prostate cancer‐specific mortality (CSM). We calculated Harrell's concordance indices (C‐index) and used competing‐risk regression to assess associations between EAU risk groups and CSM, comparing performance to multivariable models including age, clinical stage, Gleason grade, PSA doubling time and time to BCR.

**Results:**

Among the 940 patients (646 RP, 294 RT; 40.5% African American), 563 (59.9%) had low‐risk and 377 (40.1%) high‐risk BCR. The 10‐year cumulative incidence of CSM was 3.6% versus 12% for low‐risk versus high‐risk RP patients and 18.4% versus 49.5% for low‐risk versus high‐risk RT patients. EAU high‐risk BCR was associated with increased CSM in both groups (RP: HR 2.83, 95% CI 1.47–5.46; RT: HR 3.98, 95% CI 2.43–6.53). The EAU classification showed moderate discrimination (Harrell's C‐index 0.62 for RP, 0.69 for RT). Multivariable models including clinical variables demonstrated a Harrell's C‐index of 0.76 for both RP and RT.

**Conclusions:**

This first North American validation confirms moderate EAU discriminative ability. For RP patients, low 10‐year CSM in low‐risk BCR (3.6%) supports surveillance. However, low‐risk RT BCR showed substantial CSM (18.4%), exceeding high‐risk RP (12%), suggesting current criteria inadequately stratify risk after RT.

## 1 INTRODUCTION

Biochemical recurrence (BCR) of prostate cancer occurs in up to 30% of patients within 10 years of radical prostatectomy (RP) and in approximately 20%–40% after radiation therapy (RT).^1^, ^2^ However, BCR outcomes are heterogeneous: While some patients progress rapidly to metastatic disease, others experience indolent courses spanning decades.^3^ Identifying high‐risk BCR patients requiring immediate salvage intervention remains a critical clinical challenge. The European Association of Urology (EAU) proposed a simplified BCR risk stratification in 2019 to address this need.^4^ For RP, patients are classified as low‐risk (pathological Gleason grade group <4 and PSA doubling time [DT] > 12 months) or high‐risk (grade group ≥4 or PSA‐DT ≤ 12 months). For RT, analogous criteria use biopsy Gleason score and time to BCR > 18 months.^5^ Subsequently, several studies have externally validated this risk stratification: Tilki et al.^6^ in a European RP cohort, Pak et al.^7^ in an Asian RP cohort and Falagario et al.^8^ in a European cohort including both RP and RT patients. These studies demonstrated the prognostic value of this classification for metastatic progression and cancer‐specific mortality, underscoring its potential clinical utility. However, validation in North American populations remains lacking. North American cohorts differ fundamentally from European populations in racial composition and healthcare delivery, with substantially higher proportions of African American patients who experience higher prostate cancer incidence and mortality.^9^, ^10^ Given these differences, the generalizability of this European‐derived tool to North America practice requires empirical validation. We therefore aim to test the validity of the EAU BCR risk stratification in a North American institutional cohort after both RP and RT.

## 2 MATERIALS AND METHODS

### 2.1 Data source

**FIGURE 1:**
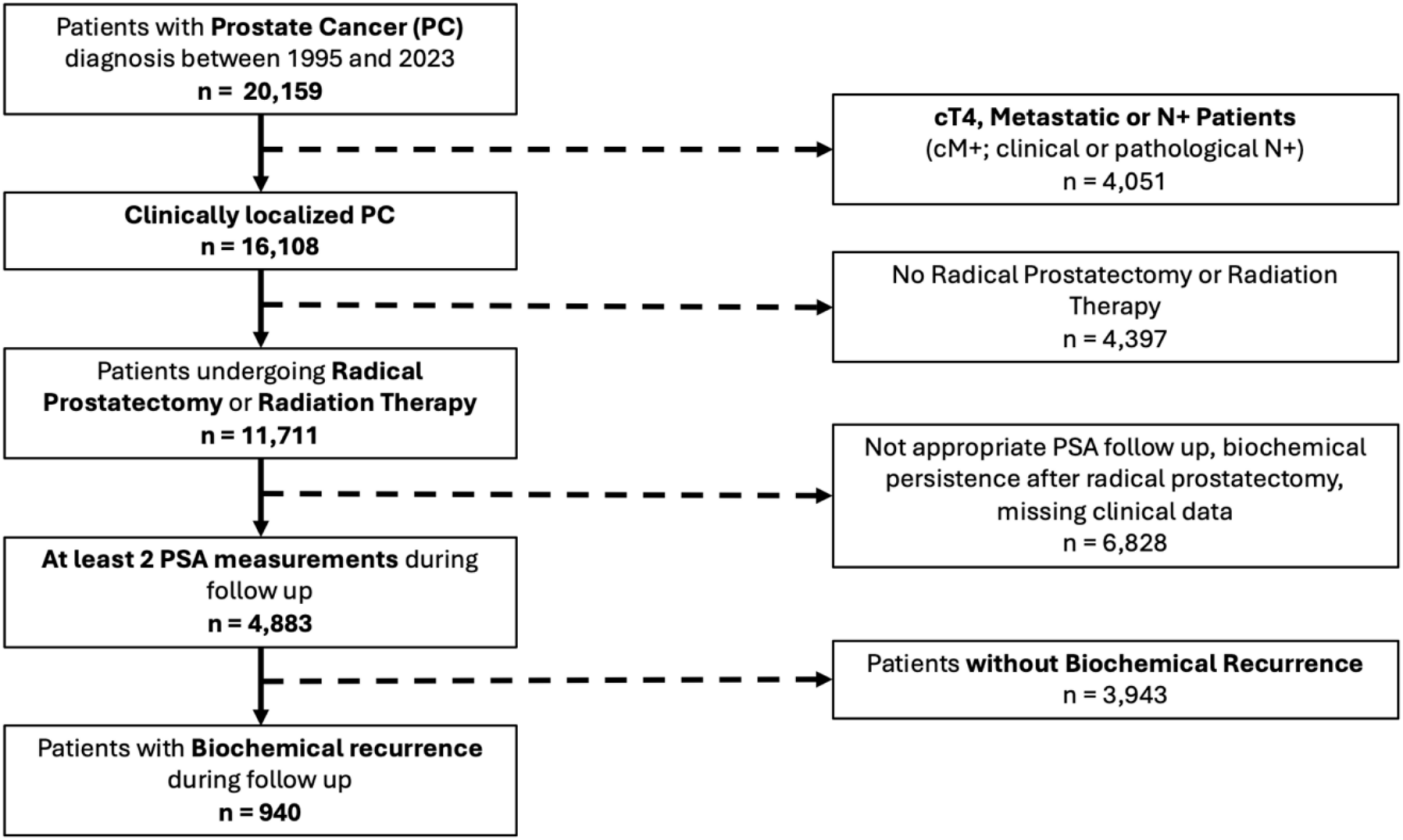
Flowchart inclusion and exclusion criteria for the final cohort of patients.

We extracted data from our institutional database of electronic medical records for all men receiving care in Henry Ford Health (HFH) between 1995 and 2022. We included all male patients who underwent either RP or RT with curative intent for localized prostate cancer (cT1‐T3) and later experienced BCR. We excluded patients with clinical or pathological lymph node involvement or with the presence of distant metastasis by the time of treatment. We included only patients with at least two PSA measurements after initial treatment. We also excluded all patients with PSA persistence after RP, defined as a PSA level that does not fall to undetectable levels after RP. Management after BCR was at the discretion of the treating physician and included observation, salvage therapy, or systemic treatment as per institutional practice at the time. Follow‐up for all patients was until death or the last available follow‐up. The study end date was set to the last available update of the HFH dataset (31 December 2022). BCR was defined as PSA ≥ 0.2 ng/mL in 2 consecutive measurements after RP and PSA rise of ≥2 ng/mL above the nadir PSA value after RT.^11^, ^12^ Our selection criteria are described in Figure 1.

### 2.2 Covariates

The following variables were extracted for each patient: age at diagnosis, Charlson comorbidity index (0, 1, 2, >2) at time of initial diagnosis (excluding prostate cancer), race (Black, White, other, unknown), area deprivation index (ADI) centile compared to US national level,^13^, ^14^, ^15^ year of diagnosis, use of androgen deprivation therapy (ADT) within initial treatment, PSA at diagnosis, clinical T stage (cT1, cT2, cT3) determined by digital rectal examination, International Society of Urological Pathology grade group (GG 1, GG 2, GG 3, GG 4, GG 5), years to BCR from surgery or RT and PSA doubling time (<= 12 months vs. >12 months) which was calculated using the two PSA values before BCR, as previously described.^16^ Patients with an undefined doubling time (difference of zero) were considered to be in the > 12 months group.

Patients were classified according to EAU BCR risk criteria as follows: after RP, low‐risk BCR was defined as PSA doubling time >12 months AND pathological ISUP grade group <4; high‐risk BCR was defined as PSA doubling time ≤12 months OR pathological ISUP grade group ≥4. After RT, low‐risk BCR was defined as time to BCR > 18 months AND biopsy ISUP grade group <4; high‐risk BCR was defined as time to BCR ≤ 18 months OR biopsy ISUP grade group ≥4.

### 2.3 Endpoints

Our endpoint is cancer specific mortality (CSM) after BCR in patients initially treated with RP or RT in a North America cohort. Cause of death was retrieved through linkage between HFH and the Michigan Vital Records.

### 2.4 Statistical analysis

Continuous variables were summarized using medians and interquartile ranges (IQR), whereas categorical variables were described as frequency and percentages. Patients were stratified by treatment type, and the groups were compared using the Kruskal–Wallis test for continuous variables. For categorical variables, chi‐square tests were performed, except when more than 20% of the expected cell counts were below 5; then Fisher's Exact test was used. For both treatment types, cumulative incidence curves were used to depict CSM, stratifying the cohort according to EAU BCR risk stratification. Statistical differences among risk categories were examined using Grey's test. Two competing‐risks regression models were built to validate the EAU BCR risk groups both for RP and RT: the first including EAU‐BCR risk groups alone and the second including age at diagnosis, clinical T stage, Gleason grade, time to BCR (continuous) from surgery or RT and PSA‐DT (categorical). Harrell's C‐index was computed to evaluate discrimination of EAU‐BCR risk groups alone versus a multivariable model in estimating CSM, using Harrell's definition of concordance probability. Confidence intervals were estimated using the delta method as described by Kang et al.^17^ Two‐sided *p*‐values <0.05 indicated statistical significance. All analyses were performed using SAS version 9.4 (SAS Institute, Cary, North Carolina). An institutional review board (IRB) waiver for informed consent was obtained before conducting this study, in accordance with institutional regulations when dealing with deidentified previously collected data.

## 3 RESULTS

Our selection criteria resulted in a population of 940 patients with BCR after initial treatment with a curative intent: 646 (68.7%) initially treated with RP and 294 (31.3%) with RT (Figure 1). The RP group was younger (median age 62 vs. 71 years, *p* < 0.001), had lower PSA at diagnosis (5.7 vs. 7.8 ng/mL, *p* = 0.002), higher proportion of Gleason grade group ≥4 disease (46.1% vs. 34.4%, *p* < 0.001) and was treated more recently (median year 2008 vs. 2006, *p* < 0.001). The RT group had higher socioeconomic disadvantage (median ADI percentile 74 vs. 53.5, *p* < 0.001). Race distribution, Charlson comorbidity index and clinical T stage were similar between groups. According to EAU stratification, 520 patients (61.7%) had low‐risk BCR and 323 (38.3%) high‐risk BCR, with higher proportion of high‐risk in the RP group (42.6% vs. 24.4%, *p* < 0.001). Clinical characteristics are presented in Table 1.

**TABLE 1:** Demographic and disease characteristics of 940 patients with biochemical recurrence, stratified by initial prostate cancer treatment^a^.

	Treatment		Total (*N* = 940)	*p*‐value
	Radical prostatectomy (*N* = 646)	Radiation therapy (*N* = 294)		
EAU BCR risk category, *n* (%)				**0.02** ^b^
EAU low risk BCR	371 (57.4%)	192 (65.3%)	563 (59.9%)	
EAU high risk BCR	275 (42.6%)	102 (34.7%)	377 (40.1%)	
Age at diagnosis				**<0.0001** ^c^
*N* (missing)	646 (0)	294 (0)	940 (0)	
Median (IQR)	62.0 (57.0, 67.0)	71.0 (65.0, 76.0)	65.0 (59.0, 70.0)	
Race, *n* (%)				0.12^b^
Black	253 (39.2%)	138 (46.9%)	391 (41.6%)	
White	345 (53.4%)	133 (45.2%)	478 (50.9%)	
Other	17 (2.6%)	7 (2.4%)	24 (2.6%)	
Unknown	31 (4.8%)	16 (5.4%)	47 (5.0%)	
CCI at diagnosis, *n* (%)				0.069^b^
0	376 (58.2%)	144 (49.0%)	520 (55.3%)	
1	127 (19.7%)	69 (23.5%)	196 (20.9%)	
2	45 (7.0%)	24 (8.2%)	69 (7.3%)	
>2	98 (15.2%)	57 (19.4%)	155 (16.5%)	
Nationwide ADI percentile				**<0.0001** ^c^
*N* (missing)	646 (0)	294 (0)	940 (0)	
Median (IQR)	53.5 (32.0, 83.0)	74.0 (50.0, 94.0)	60.0 (37.0, 88.0)	
Years to death or follow‐up from BCR				
Median (IQR)	7.1 (3.7, 12.1)	3.4 (1.6, 5.6)	5.5 (2.7, 10.3)	
Year of PCa Dx				**0.0001** ^c^
Median (IQR)	2008.0 (2001.0, 2012.0)	2006.0 (2000.0, 2010.0)	2007.0 (2000.0, 2011.0)	
PSA at diagnosis				**<0.0001** ^c^
Median (IQR)	5.7 (4.5, 8.5)	7.8 (5.2, 12.8)	6.0 (4.6, 9.6)	
Gleason grade, *n* (%)				**<0.0001** ^b^
≤ 6	34 (5.3%)	40 (13.6%)	74 (7.9%)	
3 + 4	314 (48.6%)	138 (46.9%)	452 (48.1%)	
4 + 3	175 (27.1%)	56 (19.0%)	231 (24.6%)	
8	68 (10.5%)	24 (8.2%)	92 (9.8%)	
9/10	55 (8.5%)	36 (12.2%)	91 (9.7%)	
Clinical T stage, *n* (%)				0.055^b^
cT1	475 (73.5%)	221 (75.2%)	696 (74.0%)	
cT2	164 (25.4%)	64 (21.8%)	228 (24.3%)	
cT3	7 (1.1%)	9 (3.1%)	16 (1.7%)	
Hormone therapy, *n* (%)				**<0.0001** ^b^
No hormone therapy	646 (100.0%)	197 (67.0%)	843 (89.7%)	
Hormone therapy	0 (0.0%)	97 (33.0%)	97 (10.3%)	

*Note*: EAU BCR risk classification: After radical prostatectomy, low‐risk BCR was defined as PSA doubling time >12 months AND pathological ISUP grade group <4; high‐risk BCR was defined as PSA doubling time ≤12 months OR pathological ISUP grade group ≥4. After radiation therapy, low‐risk BCR was defined as time to BCR > 18 months AND biopsy ISUP grade group <4; high‐risk BCR was defined as time to BCR ≤ 18 months OR biopsy ISUP grade group ≥4. Values in bold emphasis indicate statistically significant *p*‐values (*p* < 0.05).

^a^ All patients were node‐negative (cN0/pN0) at the time of diagnosis per study inclusion criteria.

^b^ Chi‐square *p*‐value.

^c^ Kruskal–Wallis *p*‐value.

**FIGURE 2:**
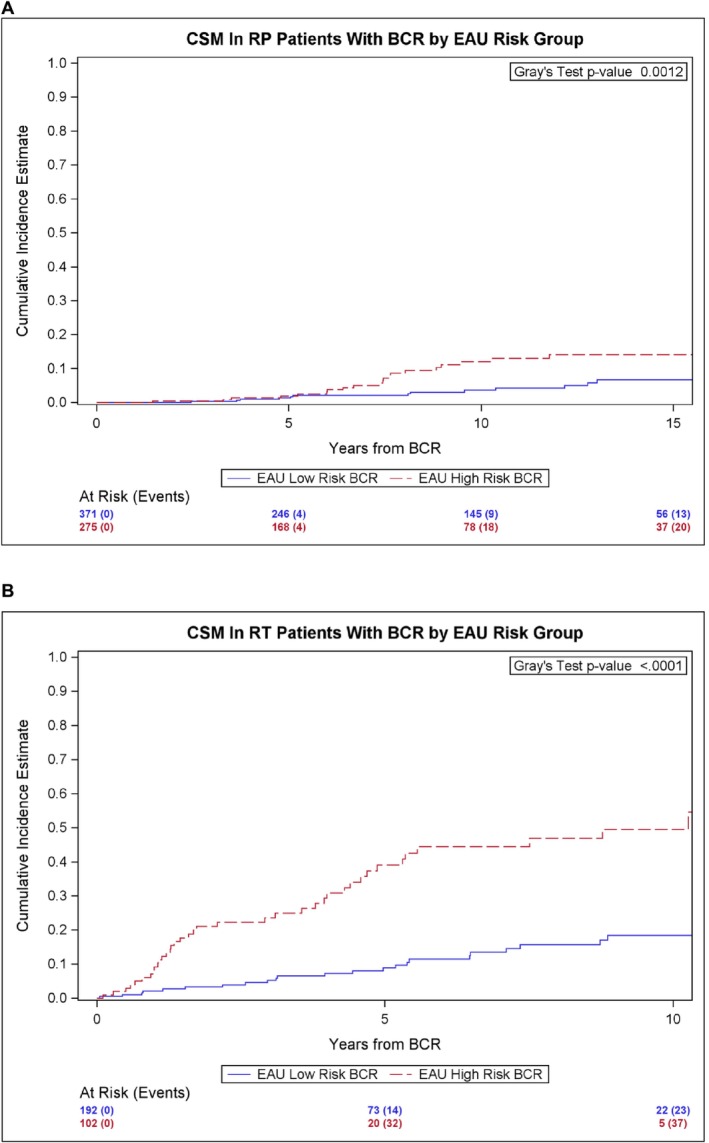
Cumulative incidence of prostate cancer‐specific mortality after biochemical recurrence stratified by EAU risk groups. (A) Patients initially treated by radical prostatectomy. (B) Patients initially treated by radiation therapy.

The 10‐year estimated CSM was 12% (95% CI 7.3%–18%) versus 3.6% (95% CI 1.7%–6.6%) for EAU high‐ versus low‐risk BCR after RP and 49.5% (95% CI 36.4%–61.3%) versus 18.4% (95% CI 11.8%–26.1%) after RT (Figure 2). On competing‐risk regression analysis, EAU high‐risk BCR was associated with increased CSM in both groups compared to the low‐risk group (RP: HR 2.83, 95% CI: 1.47–5.46, *p* = 0.002; RT: HR 3.98, 95% CI: 2.43–6.53, *p* < 0.001). The discriminative ability of the EAU risk stratification was moderate in both groups (Harrell's C‐index 0.62, 95% CI: 0.53–0.71 for RP; 0.69, 95% CI: 0.63–0.75 for RT). Multivariable models (Table 2) including age, clinical T stage, Gleason grade, PSA doubling time and time to BCR demonstrated a Harrell's C‐index of 0.76 (95% CI: 0.69–0.83) for both RP and RT (Table 3).

**TABLE 2:** Multivariable Cox regression model predicting prostate cancer specific mortality in patients with BCR after radical prostatectomy (A) and after radiation therapy (B).

(A)				
		Time unit = years from BCR		
Covariate	Level	Hazard ratio	HR *p*‐value	Type3 *p*‐value
Doubling time risk category	High risk doubling time (<=12 months)	2.06 (1.01–4.17)	**0.046**	**0.046**
	Low risk doubling time (>12 months)	‐	‐	
Age at diagnosis		1.00 (0.95–1.04)	0.8	0.8
Clinical T stage	cT2	1.31 (0.63–2.71)	0.4	0.5
	cT1	‐	‐	
Gleason grade	4 + 3	2.15 (0.91–5.11)	0.08	**<0.001**
	8	2.43 (0.77–7.69)	0.13	
	9/10	6.04 (2.26–16.15)	**<0.001**	
	≤ 6	0.00 (0.00–0.00)	**<0.001**	
	3 + 4	‐	‐	
Years to BCR from surgery		0.91 (0.76–1.10)	0.3	0.3

(B)				
		Time unit = years from BCR		
Covariate	Level	Hazard ratio	HR *p*‐value	Type3 *p*‐value
Doubling time risk category	High risk doubling time (<=12 months)	0.94 (0.43–2.06)	0.9	0.9
	Low risk doubling time (>12 months)	‐	‐	
Age at diagnosis		1.00 (0.97–1.03)	0.8	0.8
Clinical T stage	cT2	0.91 (0.51–1.63)	0.7	0.7
	cT1	‐	‐	
Gleason grade	4 + 3	1.28 (0.64–2.59)	0.4	**<0.001**
	8	6.08 (3.08–11.98)	**<0.001**	
	9/10	2.95 (1.48–5.89)	**0.002**	
	≤ 6	1.70 (0.70–4.11)	0.2	
	3 + 4	‐	‐	
Years to BCR from radiation therapy		0.74 (0.63–0.88)	**<0.001**	**<0.001**

*Note*: Number of observations in the original data set = 646. Number of observations used = 646. Number of observations in the original data set = 294. Number of observations used = 294. Values in bold emphasis indicate statistically significant *p*‐values (*p* < 0.05).

**TABLE 3:** Discriminative performance of EAU BCR risk stratification compared to multivariable model for prostate cancer‐specific mortality after radical prostatectomy (A) and after radiation therapy (B).

(A) Harrell's concordance statistic					
Source	Estimate (95% CI)	Comparable Pairs			
		Concordance	Discordance	Tied in predictor	Tied in time
EAU risk	0.62 (0.53, 0.71)	4357	1624	5292	0
MVA	0.76 (0.69, 0.83)	8581	2692	0	0

(B) Harrell's concordance statistic					
Source	Estimate (95% CI)	Comparable pairs			
		Concordance	Discordance	Tied in predictor	Tied in time
EAU risk	0.69 (0.63, 0.75)	4819	944	4418	0
MVA	0.76 (0.69, 0.83)	7778	2403	0	0

Abbreviations: EAU risk, European Association of Urology BCR risk stratification; MVA, multivariable model.

## 4 DISCUSSION

Accurate risk stratification of patients with BCR is essential to identify those who may benefit from early salvage intervention while avoiding overtreatment of indolent disease. EAU proposed a simplified BCR risk classification validated in the European and Asian cohorts after RP and in a single European study after RT. However, its performance in North American populations, characterized by different racial compositions and healthcare systems, remained unknown. In this study, we provide the first validation of this tool in a North American population after both RP and RT. Our cohort included 41.6% African American patients, reflecting a substantially different racial composition than prior European validation cohorts. The EAU classification demonstrated moderate discriminative ability for prostate cancer‐specific mortality in both treatment groups (C‐index 0.62 for RP and 0.69 for RT), comparable to previous results in the literature.^6^, ^7^, ^8^ Multivariable models incorporating PSA doubling time and time to BCR together with other clinical features demonstrated a Harrell's C‐index of 0.76 for both RP and RT. The EAU classification, as a simplified two‐category tool, offers practical clinical utility through its ease of implementation in routine practice.

Our findings in RP patients are consistent with prior EAU BCR risk stratification validations. Specifically, Tilki et al. observed in 1125 German RP patients moderate discriminative ability for CSM (C‐index 0.69).^6^ Likewise, Falagario et al. reported in 1750 Swedish RP patients a similar discrimination for CSM (C‐index 0.66).^8^ Our C‐index of 0.62 is slightly lower than the previous validations; however, confidence intervals suggest similar and moderate performance of the models. The consistent moderate discrimination across European (C‐index 0.66–0.69) and North American (C‐index 0.62) cohorts confirms that the EAU stratification provides a reasonable risk prediction for CSM in patients with BCR after RP across diverse populations. In addition, the only extra‐European validation by Pak et al. in 817 Asian RP patients confirmed significantly superior metastasis‐free survival and CSM‐free survival for low‐risk versus high‐risk BCR, despite not reporting C‐index.^7^

Validation of the EAU BCR stratification after initial RT remains extremely limited. To the best of our knowledge, Falagario et al. provided the only prior validation in RT patients predicting CSM, reporting moderate discrimination in 1191 Swedish patients (C‐index 0.69). Our study therefore represents only the second validation of this classification after RT and the first outside Europe. Our C‐index of 0.69 in 294 RT patients is consistent with this finding.

While the EAU risk stratification demonstrated similar discriminative ability after both treatment modalities, evaluation of absolute mortality rates reveals markedly different prognostic implications between RP and RT patients. Among RP patients in our cohort, 10‐year CSM was remarkably low in the low‐risk group (3.6%), with only 38 of 646 patients (5.9%) dying from prostate cancer during median 7.1‐year follow‐up after BCR. These findings align closely with prior validations: Falagario et al. reported 10‐year cancer‐specific mortality of 4% for low‐risk RP patients, while Preisser et al. documented only 9 prostate cancer deaths among 805 low‐risk BCR patients during median 54‐month follow‐up.^5^

In contrast, both our study and Falagario's reveal concerning mortality even among low‐risk RT patients. Our 10‐year CSM of 18.4% for low‐risk RT BCR patients is fivefold higher than the 3.6% observed in low‐risk RP patients and even higher than in the high‐risk RP group (12%). Falagario similarly reported substantial 10‐year mortality of 24% for low‐risk RT patients, reinforcing that even ‘low‐risk’ RT BCR carries nonnegligible mortality.^8^ For high‐risk RT patients, the risk is even more dramatic: our 10‐year cancer‐specific mortality of 49.5% underscores the aggressive biology of these recurrences.

These findings have important clinical implications for managing patients with BCR. For RP patients, the extremely low CSM in the low‐risk category (3.6% at 10 years) supports a de‐escalation in treatment as a deferred salvage intervention. This approach aligns with previous literature and guidelines and may reduce overtreatment and treatment‐related morbidity in a population with indolent disease trajectory.^4^, ^5^, ^8^ In contrast, the nonnegligible mortality observed in low‐risk RT BCR patients in our cohort (18.4% at 10 years) raises concerns about the adequacy of current EAU criteria after RT. Current EAU guidelines recommend monitoring with PSA alone for low‐risk BCR patients after RT (weak recommendation), mirroring the surveillance approach for low‐risk RP patients.^4^ However, our findings challenge the safety of this approach: Even patients classified as ‘low risk’ demonstrated mortality rates that in our population exceeded those of high‐risk RP patients. Consistent with Falagario's findings, these data suggest that the EAU low‐risk BCR definition after RT may require more stringent criteria to adequately identify truly indolent recurrences.^8^

The EAU BCR risk stratification represents the only validated tool currently available and demonstrates practical utility through its simplicity and ease of clinical implementation. However, the consistent moderate discrimination across all validations (C‐index 0.62–0.69) suggests limitations of traditional clinical parameters. Future risk stratification tools incorporating genomic classifiers (e.g. Decipher score), advanced imaging (e.g. PSMA‐PET) or tissue‐based biomarkers may enhance discriminative ability beyond what can be achieved with clinicopathologic features alone, better identifying patients requiring early intervention.^18^, ^19^, ^20^

This is the first North American validation of the EAU BCR risk stratification for both treatment modalities and only the second worldwide validation after RT. Our cohort appears different in terms of racial composition, enhancing generalizability of the EAU BCR risk stratification to slightly different populations.

Several limitations warrant consideration. Similar to prior validations, we lacked data on metastatic progression and timing, preventing assessment of the complete disease trajectory from BCR to metastasis to death.^8^ The retrospective design and reliance on electronic medical records prevented detailed ascertainment of post‐BCR management: We captured only initial treatment and lacked information on salvage treatment and use of advanced imaging (e.g., PSMA‐PET). Indeed, PSMA‐PET was not utilized for staging in this cohort, as it was not widely available during the study period (1995–2023). Future validation studies incorporating PSMA‐PET staging may provide additional prognostic information. Furthermore, patients undergoing RP versus RT differed significantly in patient‐ and disease‐related baseline characteristics, reflecting treatment selection bias inherent to observational studies, which may have influenced outcomes despite our risk‐adjustment approach. Although our report represents only the second RT external validation, our cohort (*n* = 294) had limited sample size and short median follow‐up after BCR (3.4 years). The short follow‐up was at least partially attributable to the high mortality rate in this population, which constrained statistical power for long‐term outcomes. Low event rates in RP low‐risk patients (10‐year CSM 3.6%) similarly limited discriminative assessment. Confidence intervals for Harrell's C‐index were estimated using the delta method as described by Kang et al.; while this represents the most rigorous approach currently available, no universally accepted method exists for calculating confidence intervals around concordance indices.^17^ Finally, our single‐institution design may limit generalizability, although our racially diverse patient population enhances external validity.

## 5 CONCLUSIONS

Our validation confirms that the EAU BCR risk stratification provides consistent, although moderated, discriminative ability in a North American population, with performance comparable to European and Asian cohorts. For RP patients, the extremely low cancer‐specific mortality in the low‐risk category supports surveillance as a safe strategy, avoiding overtreatment in patients with indolent disease. In contrast, low‐risk RT BCR patients demonstrate nonnegligible cancer‐specific mortality, suggesting that these patients require a better stratification and that surveillance is not necessarily a good choice in these individuals. This consistent pattern across independent cohorts strengthens confidence that the limitations observed after RT are inherent to the classification rather than population‐specific.

## AUTHOR CONTRIBUTIONS

**Carlo Silvani:** conceptualization, methodology, formal analysis, writing – original draft. **Alfonso Santangelo:** data curation, writing – review and editing. **Alex Stephens:** formal analysis, methodology. **Jack Considine:** data curation. **Shane Tinsley:** data curation. **Bassel Salka:** data curation. **Akshay Sood:** writing – review and editing. **Sebastiano Nazzani:** writing – review and editing. **Alberto Briganti:** writing – review and editing. **Andrea Salonia:** writing – review and editing. **Francesco Montorsi:** writing – review and editing, supervision. **Nicola Nicolai:** writing – review and editing, supervision. **Emanuele Montanari:** writing – review and editing, supervision. **Craig Rogers:** writing – review and editing, supervision. **Firas Abdollah:** conceptualization, supervision, writing – review and editing.

## CONFLICT OF INTEREST STATEMENT

Carlo Silvani certifies that all conflicts of interest, including specific financial interests and relationships and affiliations relevant to the subject matter or materials discussed in the manuscript (e.g., employment/affiliation, grants or funding, consultancies, honoraria, stock ownership or options, expert testimony, royalties, or patents filed, received, or pending), are the following: None.

## Data Availability

Data from the Henry Ford Health database will be made available on request in compliance with institutional and institutional review board regulations.

## References

[1] Pompe RS , Gild P , Karakiewicz PI , Bock LP , Schlomm T , Steuber T , et al. Long‐term cancer control outcomes in patients with biochemical recurrence and the impact of time from radical prostatectomy to biochemical recurrence. Prostate. 2018;78(9):676–681. 10.1002/pros.23511 29570821

[2] Lowrance W , Dreicer R , Jarrard DF , Scarpato KR , Kim SK , Kirkby E , et al. Updates to advanced prostate cancer: AUA/SUO guideline (2023). J Urol. 2023;209(6):1082–1090. 10.1097/JU.0000000000003452 37096583

[3] Pound CR , Partin AW , Eisenberger MA , Chan DW , Pearson JD , Walsh PC . Natural history of progression after PSA elevation following radical prostatectomy. JAMA. 1999;281(17):1591–1597. 10.1001/jama.281.17.1591 10235151

[4] Tilki D , van den Bergh RCN , Briers E , van den Broeck T , Brunckhorst O , Darraugh J , et al. EAU‐EANM‐ESTRO‐ESUR‐ISUP‐SIOG guidelines on prostate cancer. Part II‐2024 update: Treatment of relapsing and metastatic prostate cancer. Eur Urol. 2024;86(2):164–182. 10.1016/j.eururo.2024.04.010 38688773

[5] Preisser F , Abrams‐Pompe RS , Stelwagen PJ , Böhmer D , Zattoni F , Magli A , et al. European association of urology biochemical recurrence risk classification as a decision tool for salvage radiotherapy—A multicenter study. Eur Urol. 2024;85(2):164–170. 10.1016/j.eururo.2023.05.038 37355358

[6] Tilki D , Preisser F , Graefen M , Huland H , Pompe RS . External validation of the European association of urology biochemical recurrence risk groups to predict metastasis and mortality after radical prostatectomy in a European cohort. Eur Urol. 2019;75(6):896–900. 10.1016/j.eururo.2019.03.016 30955970

[7] Pak S , Lee DE , You D , Jeong IG , Joung JY , Lee KH , et al. Validation of the European association of urology biochemical recurrence risk groups after radical prostatectomy in an Asian cohort and suggestions for refinement. In: Urologic oncology: seminars and original investigations 39 Elsevier; 2021. 298‐e1. 10.1016/j.urolonc.2020.12.023 33579626

[8] Falagario UG , Abbadi A , Remmers S , Björnebo L , Bogdanovic D , Martini A , et al. Biochemical recurrence and risk of mortality following radiotherapy or radical prostatectomy. JAMA Netw Open. 2023;6(9):e2332900. 10.1001/jamanetworkopen.2023.32900 37695584 PMC10495864

[9] Finocchiaro A , Chiarelli G , Stephens A , Viganó S , Bertini A , Cusmano N , et al. Active surveillance for prostate cancer in “real‐world” setting: exploring racial disparities. J Racial Ethn Health Disparities. 2025;1–9. 40425976 10.1007/s40615-025-02497-4

[10] Bertini A , Tylecki A , Stephens A , Finocchiaro A , Viganò S , Cusmano N , et al. Socioeconomic disparities in prostate cancer presentation: the impact of ADI on prostate cancer stage at diagnosis. Clin Genitourin Cancer. 2025;23(6):102418. 10.1016/j.clgc.2025.102418 40961906

[11] Abramowitz MC , Li T , Buyyounouski MK , Ross E , Uzzo RG , Pollack A , et al. The Phoenix definition of biochemical failure predicts for overall survival in patients with prostate cancer. Cancer. 2008;112(1):55–60. 10.1002/cncr.23139 17968996

[12] Mir MC , Li J , Klink JC , Kattan MW , Klein EA , Stephenson AJ . Optimal definition of biochemical recurrence after radical prostatectomy depends on pathologic risk factors: identifying candidates for early salvage therapy. Eur Urol. 2014;66(2):204–210. 10.1016/j.eururo.2013.08.022 24007712

[13] Perri A , Anna T , Viganò S , et al. Socioeconomic disparities in prostate cancer treatment: the impact of area deprivation index on initial treatment type for localized prostate cancer in a North‐American state‐wide cohort. Urol Pract. 2025. 10.1097/UPJ.0000000000000940 41397111

[14] Silvani C , Santangelo A , Considine J , Tylecki A , Stephens A , Mssika A , et al. Area deprivation and cancer‐specific mortality in non‐muscle‐invasive bladder cancer: a statewide analysis. BJU Int. 2026;137(4):677–683. 10.1111/bju.70151 41556215

[15] Silvani C , Tylecki A , Santangelo A , Stephens A , Considine J , Gishto R , et al. Impact of the area deprivation index on stage at diagnosis in penile squamous cell carcinoma: a statewide cohort analysis. Cancer. 2026;132(1):e70241. 10.1002/cncr.70241 41457426

[16] Moul JW , Wu H , Sun L , McLeod DG , Amling C , Donahue T , et al. Early versus delayed hormonal therapy for prostate specific antigen only recurrence of prostate cancer after radical prostatectomy. J Urol. 2004;171(3):1141–1147. 14767288 10.1097/01.ju.0000113794.34810.d0

[17] Kang L , Chen W , Petrick NA , Gallas BD . Comparing two correlated C indices with right‐censored survival outcome: a one‐shot nonparametric approach. Stat Med. 2015;34(4):685–703. 10.1002/sim.6370 25399736 PMC4314453

[18] Spratt DE , Yousefi K , Deheshi S , Ross AE , den RB , Schaeffer EM , et al. Individual patient‐level meta‐analysis of the performance of the decipher genomic classifier in high‐risk men after prostatectomy to predict development of metastatic disease. J Clin Oncol Off J Am Soc Clin Oncol. 2017;35(18):1991–1998. 10.1200/JCO.2016.70.2811 PMC6530581 28358655

[19] Jairath NK , Dal A , Vince R , Dess R , Jackson W , Tosoian J , et al. A systematic review of the evidence for the decipher genomic classifier in prostate cancer. Eur Urol. 2021;79(3):374–383. 10.1016/j.eururo.2020.11.021 33293078

[20] Viti A , Quarta L , Zaurito P , Santangelo A , Cosenza A , Barletta F , et al. The role of genomic scores in the management of prostate cancer patients: a comprehensive narrative review. Cancer. 2025;17(14):2334. 10.3390/cancers17142334 PMC12293622 40723217

